# Magnitude and determinants of delay in diagnosis of tuberculosis patients in Ethiopia: a systematic review and meta-analysis: 2020

**DOI:** 10.1186/s13690-022-00837-y

**Published:** 2022-03-14

**Authors:** Getahun Fetensa, Desalegn Wirtu, Belachew Etana, Tadesse Tolossa, Bizuneh Wakuma

**Affiliations:** 1 Department of Nursing, School of Nursing and Midwifery, Institute of Health Sciences Wollega University, Nekemte, Ethiopia; 2 Department of Health, Behavior and Society, Institute of Health, Jimma University, Jimma, Ethiopia; 3 Department of Public Health, Institute of Health sciences, Wollega University, Nekemte, Ethiopia

**Keywords:** Tuberculosis, Delay in diagnosis, Ethiopia

## Abstract

**Background:**

Tuberculosis (TB) remains a main public health concern in the world resulting in significant morbidity and mortality as well as in Ethiopia. In Ethiopia, there are various primary studies with inconsistent findings. Delay in the diagnosis of TB is determined by different factors like the type of TB, marital status, TB-HIV co-infection, employment status, place of residence, educational status, type of first visited a health facility, and gender of the patient. This review will produce pooled evidence on delay in diagnosis and associated factors among TB that might have huge public health impacts, like unfavorable treatment outcomes, increase transmission of the disease in the community for better intervention.

**Methods:**

The presence of systematic reviews and meta-analysis on similar topics was checked and the topic was registered on PROSPERO to prevent duplication with the registration number of (CRD42020158963). Both published and unpublished studies conducted in Ethiopia from 2002 to April 1 2020 were searched thoroughly using electronic databases. Data were analyzed using STATA version 14. Heterogeneity was checked by using I^2^ and Cochrane Q test. In the presence of heterogeneity, a random effect model was employed to estimate the pooled magnitude and determinants of diagnosis delay of TB. Publication bias was checked by using the graphical funnel plot and Egger’s statistical test.

**Result:**

The Pooled magnitude of tuberculosis diagnosis delay in Ethiopia was 45.42% [95%CI 34.44, 56.40]. Residing in urban, having educational status and patients with positive serostatus were protective against TB diagnostic delay while having extra-pulmonary TB and not being married were risk factors for delaying TB diagnosis.

**Conclusion:**

TB diagnosis delays in Ethiopia are significantly high. Sociodemographic and institutional factors were significantly contributing to the delay. Therefore, national TB control programs need to identify and address gaps, barriers, and weaknesses along the entire patient care cascade, to improve appropriately.

## Background

Tuberculosis is a major public health problem worldwide [1–3]. Globally, 7.0 million new cases of TB notified in 2018 an increase from a number of the case reported 6.4 million in 2017. It is a large increase in the number of cases notified from the years 2009 to 2012 [4]. Although due to improved disease prevention and management, and service delivery; nevertheless, up to 10 million people continue to fall ill with TB every year. In 2017, a significant number of new TB cases were reported globally [1–3, 5]. Due to this, it remains a main public health concern in the world resulting in significant morbidity and mortality [1, 6–10]. As it can spread through the air as well as by personal contact with individuals affected with the disease [11]. There is success related to the incidence and mortality related to TB, its incidence was falling by 20% between 2000 and 2015. However, death-related TB remains high, with 1.8 million deaths in 2015 alone [12]. Forty percent of TB cases in Africa are under-diagnosed or under-reported [13]. Ethiopia accounts for 3% of the annually 3 million missed people with TB by the global health system, which was estimated to be 35%(56,164) of incident TB cases were missed in 2016 [6]. Tuberculosis is the main reason for illness, the third cause of hospital admission (after deliveries and malaria), and the second cause of death in Ethiopia, after malaria [10].

Ethiopia could not achieve an on-time diagnosis. The problem is contributed from different factors like inadequacy of resources for TB case finding such as a shortage of healthcare providers, inadequate basic infrastructure, and inadequate diagnostic equipment and supplies [13, 14].

It is important that healthcare providers have to be sufficiently familiar with the basic principles of TB diagnosis and care, to ensure early case identification and prompt referral to specialized centers for treatment initiation and follow-up [15].

Delays in diagnosis of tuberculosis patients have several public health impacts in the patients as well as in the community and country as a whole [1, 16]. It is significantly higher within extra-pulmonary than among those with pulmonary TB [17].

In Ethiopia evidence indicates that the proportion of tuberculosis patients who had delayed diagnosis was 59.9% [16, 18]. Delays in diagnosis were significantly longer for patients who attended a non-TB facility first, and even longer for those who failed to follow the physician’s recommendation to seek care at a TB facility [1, 19]. Delays in tuberculosis (TB) diagnosis is the main barrier to the effective management of the disease [20]. World Health Organization had made different efforts to narrow gaps in TB detection and treatment, in 2018 through an initiative called to find to achieve estimates that at least 30 million people will be eligible for TB preventive treatment between 2018 and 2022 [21].

Diagnosis of Tuberculosis employs the use of various diagnostic methods. Under certain circumstances this may not be achieved due to either lack of literate workforce or facility in 2016, an estimated 35% of incident TB cases were missed in Ethiopia [6, 22]. On another way, most of the laboratory tests employed in Ethiopia are based on direct smear microscopy, which is insensitive and can only detect 36% of tuberculosis cases which can contribute to delay of TB to some extent [23].

Delay in the diagnosis of TB, which leads to underreporting of detected cases, and under-diagnosis. This can mislead policymakers and clinicians in managing tuberculosis [1, 3]. In another way, fear of stigma towards TB can also lead to delay in health care seeking [24, 25]. In-country like Ethiopia, the effect is very high as the point prevalence estimate (per 100,000) of undiagnosed smear-positive Pulmonary Tuberculosis (PTB) included was 79.7% [26]. TB prevalence is not only the factor to cause to cavitation of the lung tissue, but also delays in healthcare-seeking can do so. Ninety percent of lung degradation occurs with a delay of more than 30 days [27, 28].

Studies have indicated different factors associated with a diagnosis of TB. Even if some evidence indicates, the burden of TB is higher in men than in women [29]. However another result reveals that the female gender is one of independent predictors for not seeking diagnosis and treatment for tuberculosis as a mixed-method study from India indicates [19, 21, 30–32]. Rural areas patients were more likely to have experienced delayed diagnosis [33].

Furthermore, characteristics of TB patient and characteristics of the diseases also associated with timely diagnosis of TB. Study indicated that family and patient knowledge about the disease decreases a delay in diagnosis [24, 34]. In another way, new patients were about three times more likely to come late for TB diagnosis as compared with those who had the previous history of treatment [16]. The result from England also reveals that extra-pulmonary TB disease is significantly associated with longer diagnostic delays [32]. Smear-positive patients experienced longer delays in seeking care but shorter diagnostic delays [19].

There are primary studies in Ethiopia that show the level of diagnosis delay with inconsistent results and factors. However, there is a need to pool the finding of these studies for decision-makers and health care programs to indicate the over level of delay in the country. Therefore, this study was done to review existing evidence through systemic review and pool the delay in magnitude using systemic review and meta-analysis.

## Methods

### Search strategy

This systemic review and meta-analysis were conducted to assess delays in the diagnosis of tuberculosis patients and determinant factors in Ethiopia. We checked the presence of systematic reviews and meta-analysis on this topic and the topic was registered on PROSPERO to prevent duplication (CRD42020158963). Both published and unpublished studies conducted from 2002 to April 1, 2020 were searched thoroughly using electronic databases such as Medline, Embase, Hinari, Pub Med, Cochrane library, the Web of Science, and Google Scholar using the key terms “Tuberculosis” “magnitude of TB” prevalence of TB” TB treatment delay” “delay in TB diagnosis” “associated factors”, determinants”, Ethiopia”. To find unpublished papers, some research centers, including the Digital Library of universities in Ethiopia were searched. All articles published and unpublished until April 1, 2020, were as included.

Pre-defined search terms were used to enable a comprehensive search strategy that included all the relevant studies. All fields within records and Medical Subject Headings (MeSH terms) were used to expand the search in advanced Pub Med search. The search strategy was prepared and modified for the various databases using important Boolean operators with initial keywords *“*(((((prevalence, TB treatment/diagnosis delay) OR magnitude, TB treatment/diagnosis delay) AND associated factors, TB treatment/diagnosis delay) OR determinants, TB treatment/diagnosis delay) AND TB treatment/diagnosis delay) AND Ethiopia*}.* The meta-analysis was reported using Preferred Reporting Items for Systematic Reviews and Meta-Analyses (PRISMA) guideline [35]. Literature were downloaded to Endnote (version X7.2,) to maintain and manage citation and facilitate the review process.

### Selection and eligibility criteria

**Fig. 1:**
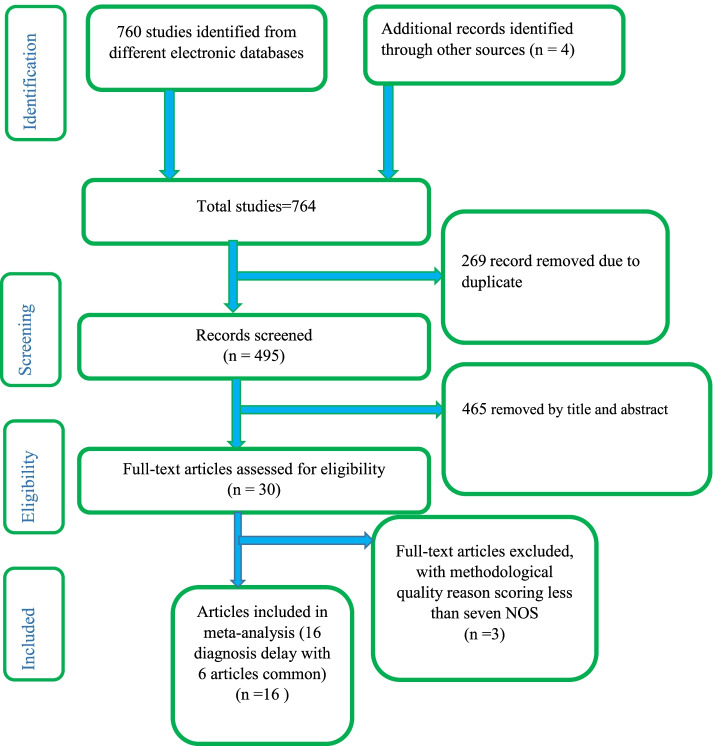
PRISMA flow diagram of included studies in the systematic review and meta-analysis of determinants of delay in diagnosis and treatments of tuberculosis patients in Ethiopia, 2020: systematic review and Meta-analysis

This systematic review and meta-analysis included studies that were conducted on the delay in the diagnosis of tuberculosis patients and determinant factors. This review considered all observational study designs, which reported the delayed diagnosis and associated factors of tuberculosis that were written in the English language. This review included studies conducted only in Ethiopia and a master’s thesis from grey literature. There were no limits on the studies by sample size. We had excluded articles, with studies with low-quality assessment scale due to methodological problems. Studies with multi-drug resistant TB were excluded. Studies conducted before 2002 were excluded due to the fear of result distortion of finding (Fig. 1).

### Outcome measurement

This systematic review and meta-analysis have four main outcomes. The primary outcome of review was the delay in diagnosis of tuberculosis, which was estimated as the total number of patients with delay in TB diagnosis cases divided by the total number of TB patients multiplied by 100%. The other outcomes was determinant factors of delay diagnosis among tuberculosis patients, which was determined in the form of odds ratio and calculated, based on the binary outcome from the included primary studies. The major factors were identified after reviewing all primary articles.

### Quality assessment and data extraction

The citation management software (Endnote version X7.2) was used to combine database search results and to remove duplicate articles manually. Newcastle-Ottawa Scale (NOS) adapted for cross-sectional studies was used for quality assessment. Data were extracted by GF and checked by TT using standardized data extraction checklists on Microsoft excel. For the first outcomes (delay in diagnosis), the data extraction checklist included the title, author name, year of publication, region, study design, sample size, number of the subject with the outcome. For the third and fourth outcomes (determinant factors), data were extracted in a format of two-by-two tables, and then the log odds ratio for each factor was calculated based on the findings of the original studies. Discrepancies between two independent reviewers were resolved by after discussion for the consensus.

### Statistical analysis and synthesis

A systematic review was conducted to compare and contrast as well as to describe results from the primary studies. While the meta-analysis data were analyzed using STATA version 14. The logarithm and standard error of the odds ratio (OR) for each included study were generated using the “generate” command in STATA.

### Assessment of heterogeneity

Heterogeneity was evaluated using the Q test and inverse variance index (I^2^). The random effect model was used for analyses to estimate the pooled effect of delay in diagnosis of tuberculosis patients.

### Publication bias

A funnel plot of asymmetry was used to check the presence of publication bias. Furthermore, Egger statistical test was used to check the statistical significance of publication bias, and the I-squared statistic (I2 = 100% × (Q-df)/Q). For the Q test, a *P*-value of 0.10 or less was considered statistically significant, indicating marked heterogeneity among studies. I2 is a relative measure. It compares the variation due to heterogeneity (τ 2) to the total amount of variation in a ‘typical’ study (τ 2 + ∈ 2), where ∈ is the standard error of a typical study of the review [36]. For subgroup analysis, the heterogeneity within groups was tested, using the same statistical methods. A subgroup analysis was conducted by region (the area where studies were conducted).

### Ethical consideration

Not applicable. Because the author used articles that were already secured ethical issues in Ethiopia.

### Operational definition and definition of terms

**Fig. 2:**
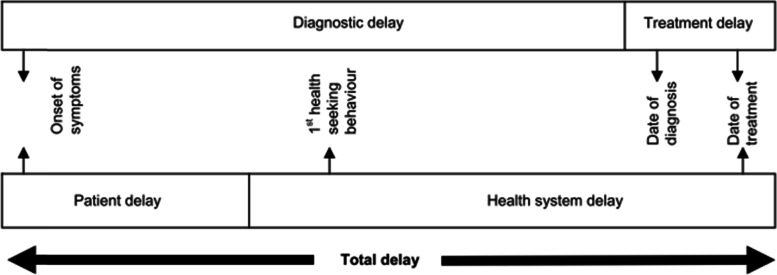
Operational definition used for TB diagnostic delay obtained from literature review Ethiopia, 2020: systematic review and meta-analysis [10]. Legend: Y-axis indicate the authors of the primary studies and year of publication. X-papers contribution within the study which is indicated in figure

Diagnostic delay: time interval between the onset of symptoms and labeling of the patient as a tuberculosis patient (tuberculosis diagnosis) [4, 37] (Fig. 2).

Patient delay will be measured using patients’ recall of first TB symptoms as the starting point, (usually described as the onset of a persistent cough) and the date of the first health facility consultation as the endpoint [4, 20, 37].

Health care system delay: time interval between the date of health-seeking behavior at a health care provider and the initiation of anti-tuberculosis treatment [4, 37].

## Result

### Result for delayed diagnosis of tuberculosis in Ethiopia

**Fig. 3:**
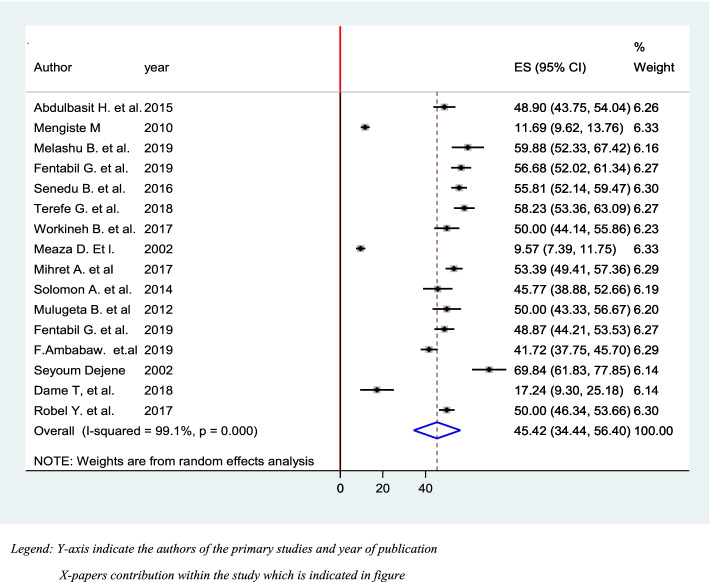
Pooled prevalence of diagnostic delay among tuberculosis patients in Ethiopia, 2020: systematic review and Meta-analysis. Legend X-axis indicates prevalence. Y-axis indicates the standard error of prevalence

**Fig. 4:**
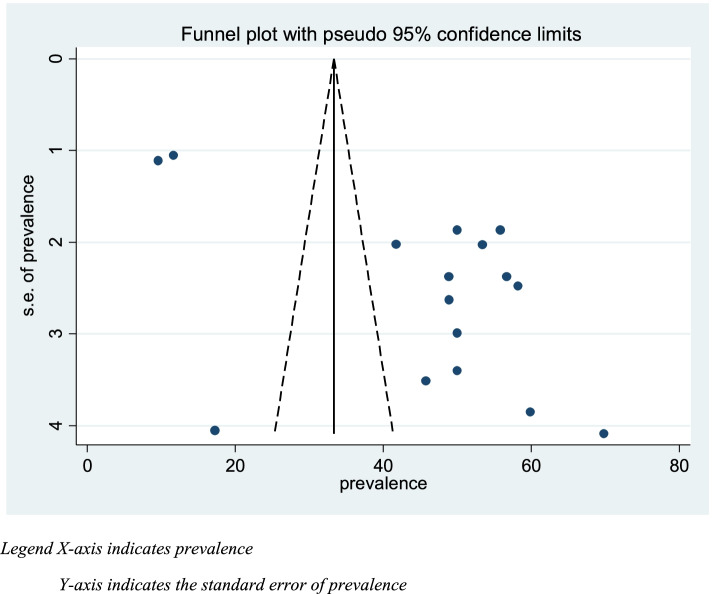
Funnel plot for prevalence of Diagnosis delay among patients with Tuberculosis in Ethiopia 2020: systematic review and Meta-analysis. Legend: X-axis indicates. Y-axis indicate authors list and year of publication

A systematic search of electronic databases and library catalogs identified a total of 764 published articles and four unpublished studies. A total of 16 studies with a sample size of 6948 were included to determine the pooled prevalence of diagnostic delay of tuberculosis patients in Ethiopia. Of these studies five of them were conducted in the Amhara regional state [38–42] and three of them in the Oromia regional state [43–45]. Another of two studies were conducted in SNNPE [42, 46] one in Tigrai regional state [47], one in Harari Regional state [48], One in Somali regional state [49], one study in Addis Ababa city administration, one study conducted in two regions of the country and one in Afar regional state [50] (Fig. 1). Accordingly, the pooled prevalence of tuberculosis diagnosis delay in Ethiopia is 45.42% at [95%CI 34.44, 56.40] (Fig. 3. The presence of publication bias was checked and indicated the funnel plot (Fig. 4). The median diagnosis delay for the included study was 45 days. A maximum prevalence was observed in the Somali region 68.84 at [95% CI 61.83, 77.85] [49] while the minimum prevalence was in Addis Ababa city 9.57 at [95%CI 7.39,11.75] [51] (Table 1).

**Table 1:** Abstraction of studies included in determining the prevalence of diagnostic Delay among Tuberculosis patients in Ethiopia, 2020: systematic review and Meta-analysis

no	Author	Year of publication	study design	Region	Study Area	sample size	Prevalence	Delayed diagnosis cases	Median delay in days	NOS
1	Abdulbasit H. et al. [52]	2015	Cross-sectional	Oromia	Asella, Robe and Abomsa of Arsi zone	362	48.9(43.75,54.01)	177	40	10
2	Mengiste M. et al. [53]	2010	Cross-sectional	Tigray	Ten districts of Tigray region	924	11.69(9.62,13.76)	108	30	9
3	Melashu B. et al. [54]	2019	Cross-sectional	Amhara	North shoa	162	59.88(52.33,67.42)	97	53.2	9
4	Fentabil G. et al. [31]	2019	Cross-sectional	Somali	Four Hospitals in Somali	434	56.68(52.02,61.34)	246	49	9
5	Senedu B. et al. [55]	2016	Cross-sectional	Amhara	West Gojjam	706	55.81(52.14,59.47)	394	22	10
6	Terefe G. et al. [56]	2018	Cross-sectional	SNNPE	Hadiya Zone	395	58.23(53.36,63.09)	230	30	8
7	Workineh B. et al. [57]	2017	Cross-sectional	Harar	Harar town	280	50.00(44.14,55.86)	140	21	7
8	Meaza D. et al. [51]	2002	Cross-sectional	AA	Addis Ababa city	700	9.57(7.39,11.75)	67	60	10
9	Mihret A. et al. [58]	2017	Cross-sectional	Amhara		605	53.39(49.41,57.36)	323	45	8
10	Solomon A. et al. [59]	2014	Cross-sectional	Amhara	Bahirdar City	201	45.77(38.88,52.67)	92	27	10
11	Mulugeta B. et al. [50]	2012	Cross-sectional	Afar		216	50.00(45.33,56.67)	108	70.5	8
12	Fentabil G. et al. [28]	2019	case-control	Somali		442	48.87(44.21,53.53)	216	50	9
13	F.Ambabaw. et al. [60]	2019	Cross-sectional	SNNPE and Amhara		592	41.72(37.75,45.70)	247	84	9
14	Seyoum Dejen e[61]	2002	Cross-sectional	Somali		126	68.84(61.83,77.85)	88	171	7
15	Dame T. et.al [62]	2018	Cross-sectional	Oromia	Mettu Town	87	17.24(9.30,25.18)	15	NR	7
16	Robel Y. et al. [63]	2017	Case-control	Oromia	Arsi zone, Seru	8716	50.00946.34,53.66)	358	15	9

*NOS* Newcastle-Ottawa Scale

### Sensitivity analysis

**Fig. 5:**
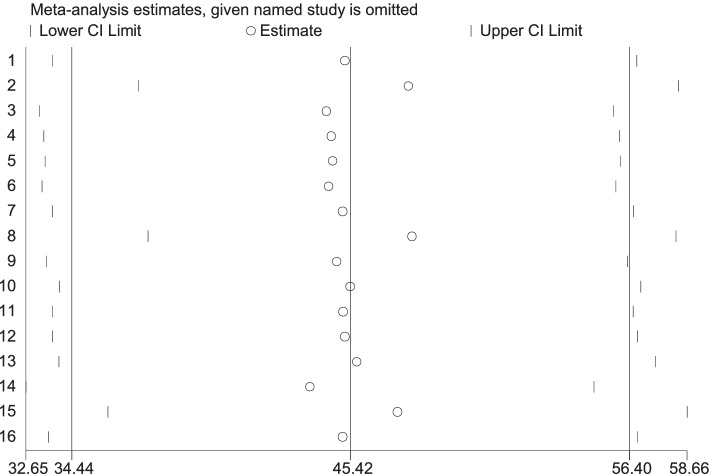
Sensitivity analysis on TB diagnosis delay in Ethiopia 2020: systematic review and Meta-analysis. Legend: X-axis indicates the estimated contribution of individual papers and within the region. Y-Authors of the primary studies and years of publication

Sensitivity analysis was looked for variables included to identify TB diagnostic delay for identifying the presence of any outliers of a single study influence on the overall meta-analysis, it was conducted using a random-effects model, and the result reveals that there was no evidence for the effect of a single study on the overall meta-analysis result (Fig. 5).

### Subgroup analysis

**Fig. 6:**
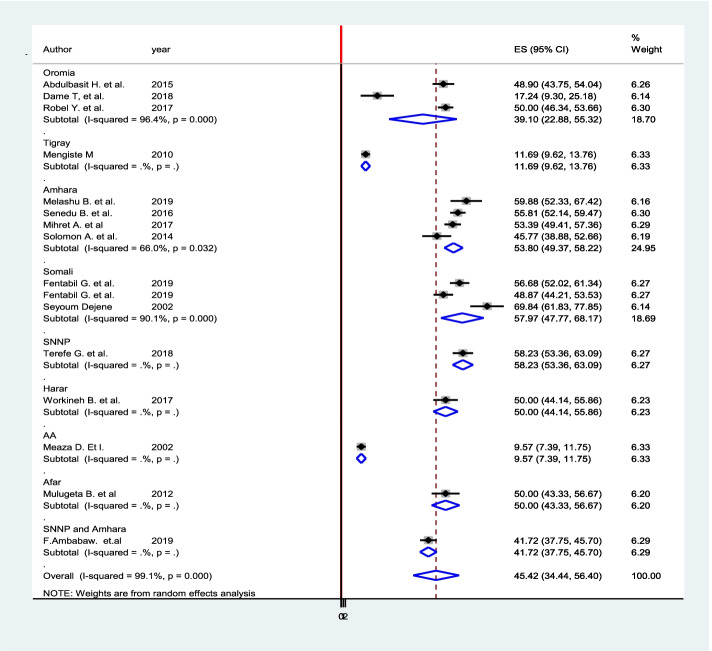
Subgroup analysis based on the region in which the study was conducted on TB diagnosis delay in Ethiopia 2020: systematic review and Meta-analysis. Legends X-axis strength of each paper within the study. Y-axis Authors and year of publications

Subgroup analysis was conducted based on the regions in which the studies were conducted to reduce the possible random disparity between studies. The maximum prevalence was observed in SNNP 58.23 at 95% CI (53.36, 63.09), while the minimum prevalence was observed in Addis Ababa 9.57 at 95% CI (7.39, 11.75) (Fig. 6).

### Determinants of delay in diagnosis

#### Type of TB and delay in diagnosis of TB

**Fig. 7:**
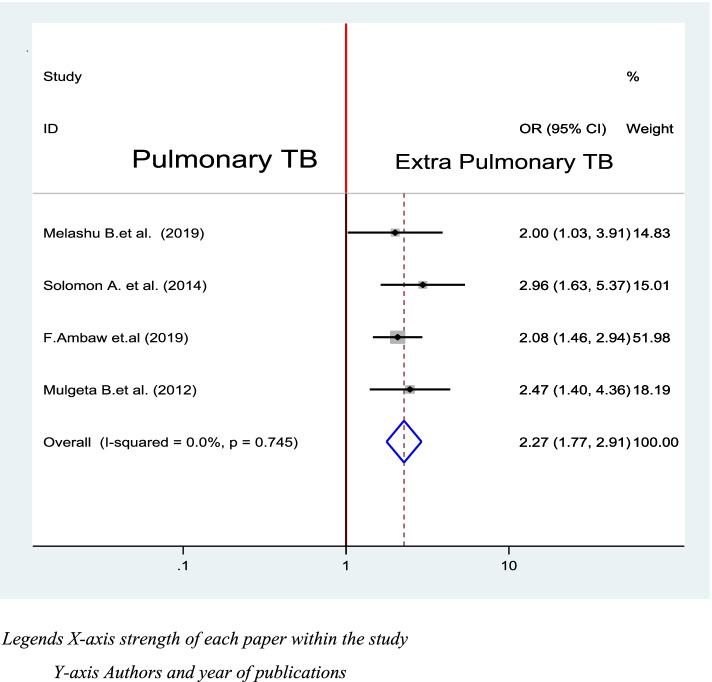
Association between Diagnosis delay and Type of Tuberculosis in Ethiopia 2020: systematic review and Meta-analysis. Legends X-axis strength of each paper within the study. Y- axis Authors and year of publications

Patients with extra-pulmonary TB are 2.27 more likely to delay diagnosis of tuberculosis when compared with patients with pulmonary TB at (OR = 2.27, CI: 1.77, 2.91). Four studies out of 16 studies were included to look association between type of TB and delay in diagnosis [38, 39, 42, 50]. All studies were significantly associated independently (Fig. 7).

### Marital status and delay in the diagnosis of TB

**Fig. 8:**
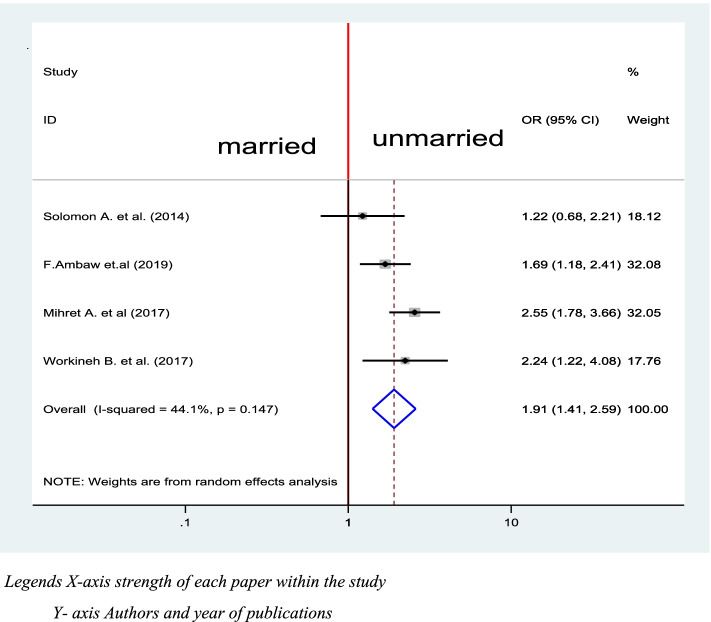
Association between Marital status and Delay in diagnosis of TB in Ethiopia 2020: systematic review and Meta-analysis. Legends X-axis strength of each paper within the study. Y- axis Authors and year of publications

Four studies were included to compute the association between marital status and delay in the diagnosis of tuberculosis for the final meta-analysis [39, 41, 42, 48]. A random-effect model was used to estimate the pooled association between marital status and delay in diagnosis of tuberculosis (I^2^ 44.1%, *P* = value <0.147). Among included studies, three of them have significant association with delay in diagnosis of TB [41, 42, 48] while one of the included did not have significant association [39]. The pooled result of the analysis indicates that unmarried individuals were 1.91 more likely to delay the diagnosis of tuberculosis (OR = 1.91, CI: 1.41, 2.59) (Fig. 8).

### HIV status and delay in the diagnosis of TB

**Fig. 9:**
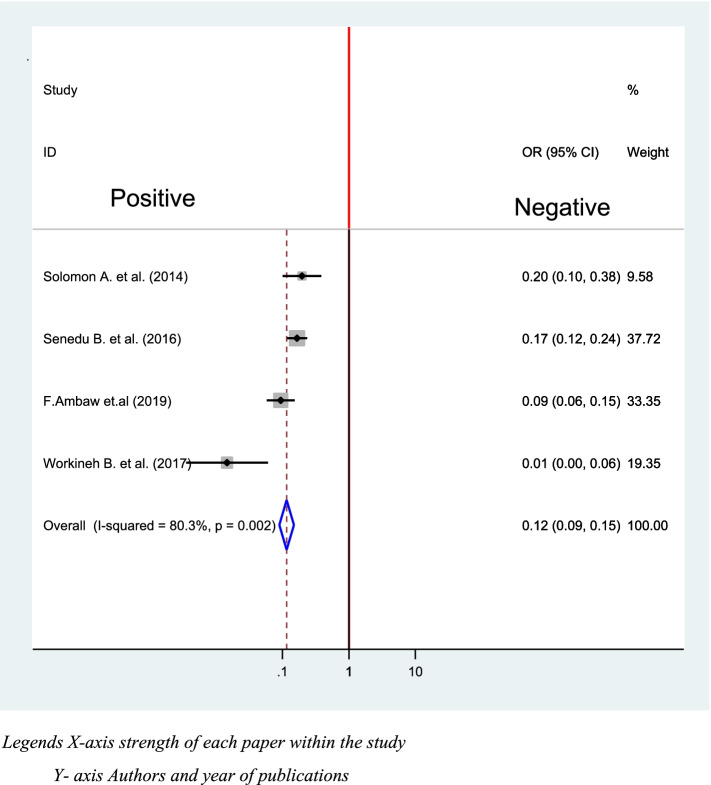
Association between HIV status and delay in diagnosis of TB in Ethiopia 2020: systematic review and Meta-analysis. Legends X-axis strength of each paper within the study. Y-axis Authors and year of publications

Four studies were utilized to analyze association between HIV Sero- status and Delay in the diagnosis of TB [39, 40, 42, 48]. The pooled indicates that the odds of not delaying TB diagnosis is 88% more likely for people living positively when compared with their counterparts 95% (OR = 0.12, CI: 0.09, 0.15) (Fig. 9).

### Patients employment and delay in diagnosis of TB

**Fig. 10:**
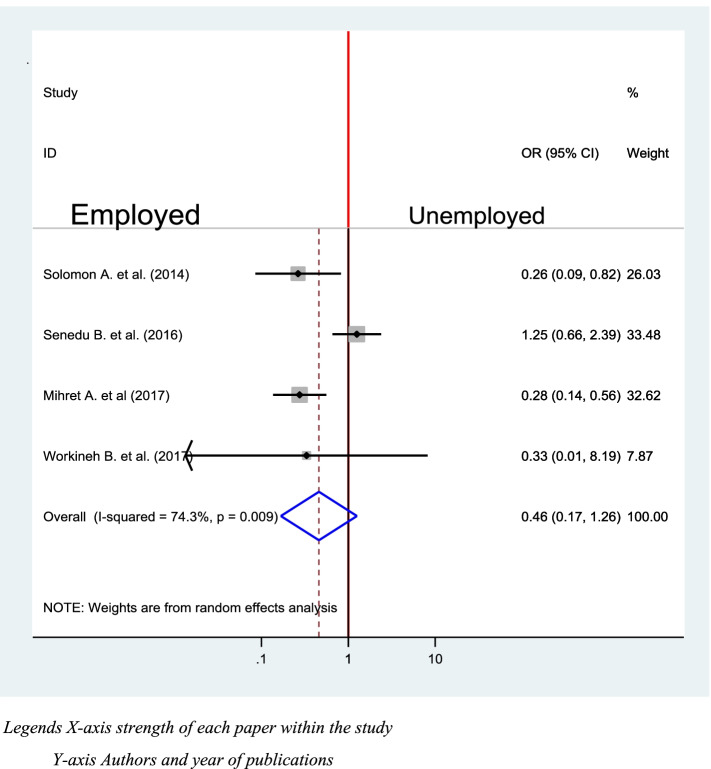
Association between employment status and delay in diagnosis of TB in Ethiopia 2020: systematic review and Meta-analysis. Legends X-axis strength of each paper within the study. Y- axis Authors and year of publications

Four studies were included to check the association between employment status and delay in diagnosis of TB [39–41, 48]. Two of the included were not significantly associated with a delay in diagnosis of TB [40, 48], but two of them were significantly associated [39, 41]. However, the pooled reveals that there is no significant association between delay in diagnosis of TB and employment status at (OR = 0.46, 95%CI (0.17, 1.26) (Fig. 10).

### Place of residence and delay in diagnosis of TB

**Fig. 11:**
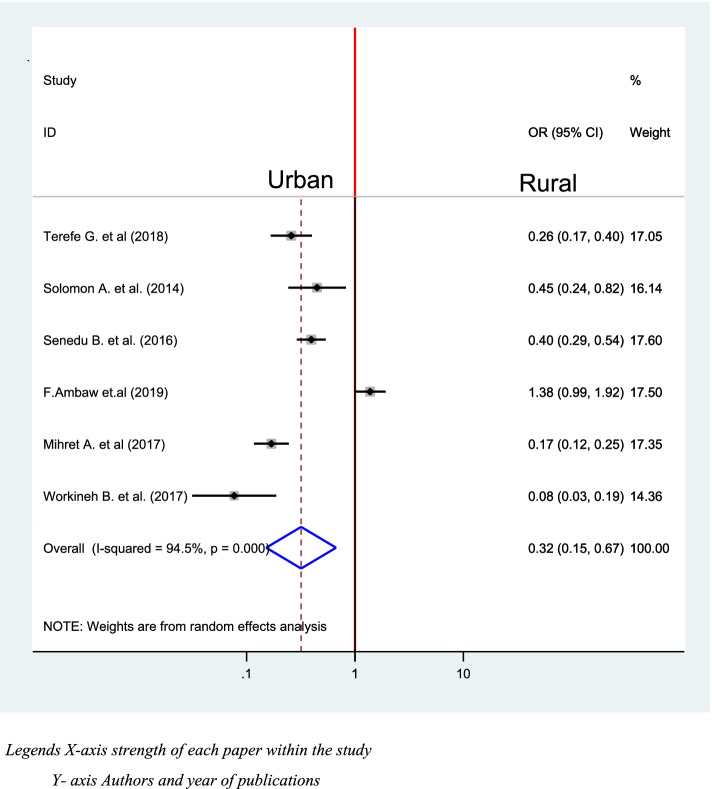
Association between place of residency and diagnostic delay of TB in Ethiopia 2020: systematic review and Meta-analysis. Legends X-axis strength of each paper within the study. Y- axis Authors and year of publications

Six studies were included to check the association between place of residency and Delay in the diagnosis of TB [39–42, 46, 48]. One of the included studies was not significantly associated [42], While the rest five of them were significantly associated. The pooled effect indicates urban residents were 68% times more likely to not delay a diagnosis of TB than Rural residents (OR = 0.32, 95% CI: 0.15, 0.67) (Fig. 11).

### Educational status and delay in diagnosis of TB

**Fig. 12:**
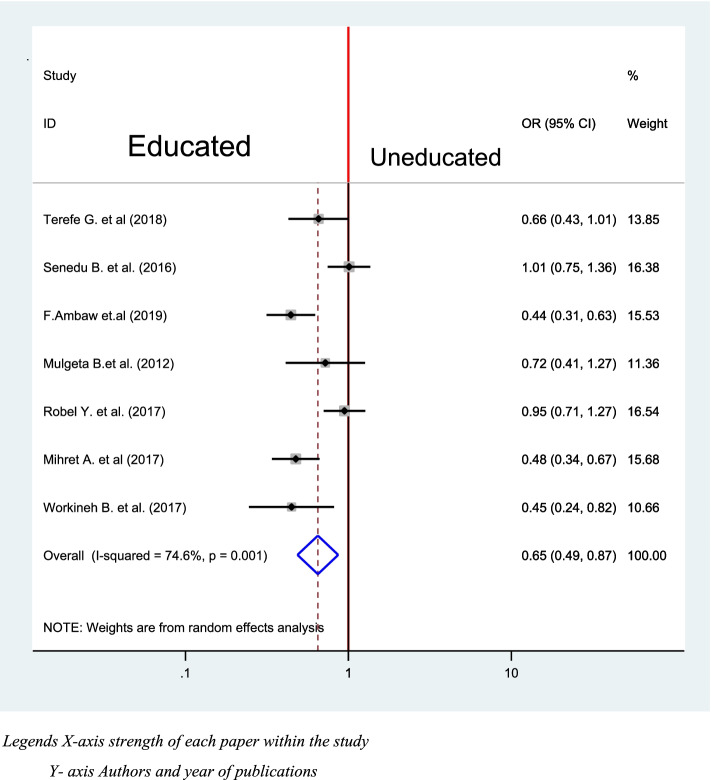
Association between Educational status and delay in diagnosis of TB in Ethiopia 2020: systematic review and Meta-analysis. Legends X-axis strength of each paper within the study. Y- axis Authors and year of publications

Seven studies from all were included to check the association between educational status and Delay in the diagnosis of TB [40–42, 45, 46, 48, 50]. Three of the studies were significantly associated [41, 42, 48], while four of them were not [40, 45, 46, 50]. The pooled result of the analysis indicates that being educated lowers delay in diagnosis of TB by 35% times when compared with uneducated patients (OR = 0.65, 95% CI: 0.49, 0.87) (Fig. 12).

### First visited health facility and delay in diagnosis of TB

**Fig. 13:**
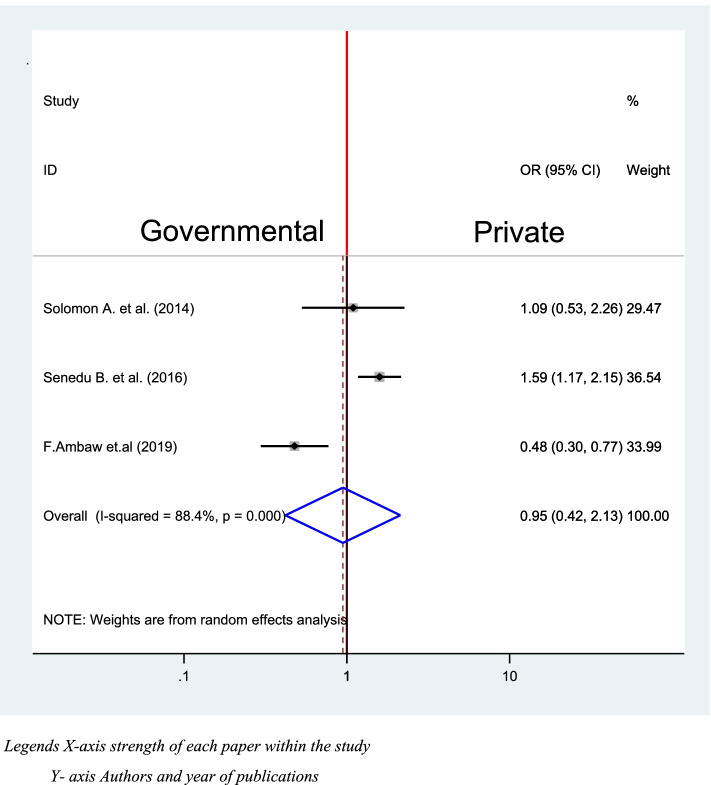
Association between first visited health facility and delay in diagnosis of TB in Ethiopia 2020: systematic review and Meta-analysis. Legends X-axis strength of each paper within the study. Y- axis Authors and year of publications

Three studies were identified to assess the association between first visited health facilities and delay in diagnosis of TB [39, 40, 42]. Of the included studies two studies were significantly associated with a delay in diagnosis of TB [40, 42], while one of the studies [39] and the pooled included studies were not significantly associated with (OR = 0.95, 95% (0.42, 2.13) (Fig. 13).

### Sex of respondents and delay in diagnosis of TB

**Fig. 14:**
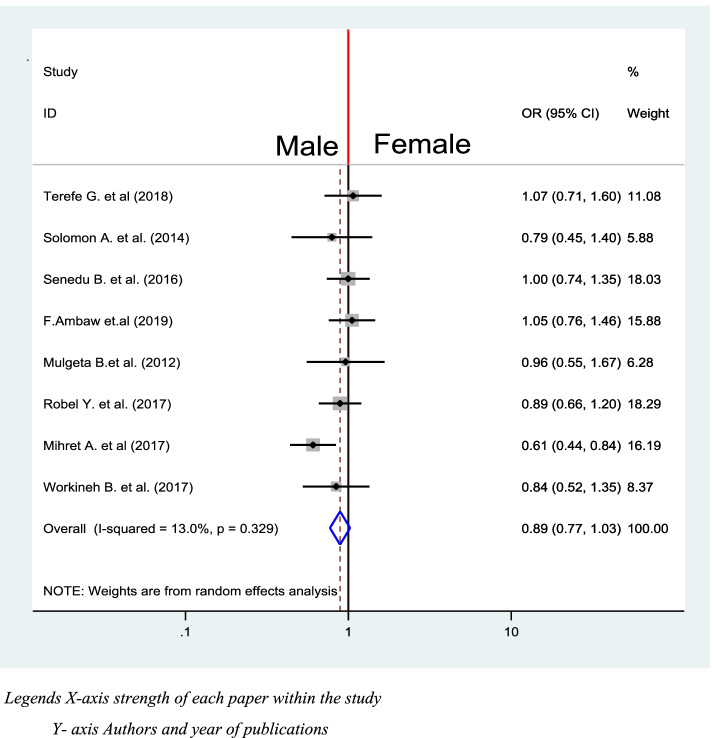
Association between sex of respondent and Delay in diagnosis of TB in Ethiopia 2020: systematic review and Meta-analysis

Half of the identified studies were included to assess the association between sex and delay in diagnosis of TB [39–42, 45, 46, 48, 50]. Out of all studies, only one of them reveals significant association between delay in diagnosis and treatment of [41]. The pooled revealed there is no significant association between smear sex of respondents and delay in diagnosis of TB (OR = 0.89, 95% CI: 0.77, 1.03) (Fig. 14).

## Discussion

The Pooled prevalence of tuberculosis diagnosis delay in Ethiopia was 45.42%. This result is higher than the findings from Shenzhen of China 27.8% [19] and England (34.0%) [32]. This significant prevalence of TB diagnostic delay implies as innovative diagnostic platforms for effective response in Ethiopia are needed urgently [64], As it can speed up disease prognosis at the individual level and increase communicability of disease within the society [54] However, this result it is lower than the study results from Uganda [59], Kenya [55], Pakistan [58], and Brazil, Sao Paulo [60]. This is because accessibility and health care facility of this country is different.

In another way in the current study evidence suggested that the median diagnosis delay was 45 days. Which is lower than the study result from, Angola 64 days [52], While it is lower than the study result from Kenya 37.3 days [62], India 31 days [63].

Improving current diagnostic and therapeutic practices will supplement the WHO end-TB Strategy launched post-201 5[15].

In the current study, patients with extra-pulmonary TB are 2.27 more likely to delay diagnosis of tuberculosis as compared with patients with Pulmonary TB at (OR = 2.27, CI: 1.77, 2.91). This result is comparable to other similar studies that tuberculosis patients with extrapulmonary site involvements were more likely to delay in seeking health service as compared with patients with only pulmonary site involvement [16, 53, 56]. This is consistent with study results from England [32] and Italy [17]. This implies that TB patients with extra-pulmonary TB need special attention from policymakers and clinicians because the time of delay in diagnosis increase with increases of pulmonary cavitation [16]. This finding strongly indicates that health professionals have to give special attention for patients with extrapulmonary TB to prevent further complications and transmission.

This study reveals that residing in the urban area lowers 68% risk of in delay diagnosis of TB than rural residents (OR = 0.32, 95% CI: 0.15, 0.67). This is consistent with studies conducted in Pakistan, England, and Chinese which states that patients from rural areas were more likely to have experienced delaying diagnosis [32, 33, 57]. This can be justified, as there is a difference in access to health care and awareness of early seeking health facility.

According to the current study, being educated lowers delay in diagnosis of TB by 35% when compared with counterparts (OR = 0.65, 95% CI: 0.49, 0.87). This is consistent with the study result from Sao Paulo, Brazil [60]. This implies that educated peoples have good awareness in early diagnosis seeking and play a pivotal role in the prevention of communicable disease.

The result of analysis indicates that unmarried individuals were 1.91 more likely to delay diagnosis of tuberculosis (OR = 1.91, 95% CI: 1.41, 2.59). This might be due to the reality that lack of social support may lead to delay in health care seeking which can lead to delay in diagnosis [11].

In the current study, people living positively were 88% less likely not to delay in diagnosis of TB when compare with their counterparts (OR = 0.12, 95%CI: 0.09, 0.15). This is due to the fact that the WHO and TB Strategy gives a framework for TB and HIV programs to unite with each other and with other sectors to attain the sustainable development goals contributes for early detection of the case among people living positively [61].

### Study strengths and limitations

The Study have considered different inconsistent data from different part of Ethiopia, to come up with pooled data. On the top if this researchers have under gone robust analysis, which can be considered as strength. As the review was based only on articles conducted in the English language, it might overlook other articles. As well there is no single study with a diagnosis from two regions of Ethiopia (Gambella National Regional state and Benishangul Gumuz national regional state) that might lead to under representation of the region within the country.

## Conclusions

The pooled result indicates that delay in TB diagnosis was still significantly high in Ethiopia. Factors like urban residence, having certain education and positive HIV serostatus were protective factors for TB patients’ delay in diagnosis. While having extrapulmonary TB unmarried and unmarried marital status were a risk factor for delayed diagnosis of TB.

### For health facilities

- In a communicable disease like TB control program early diagnosis important is important in the prevention and control of TB. Strengthening health facilities for diagnosis of TB for patients indicating signs and symptoms during the visit is mandatory.

-Health facilities have to include education about TB for all patients seeking service as knowledge about TB has positive influences on diagnosis, paying special attention to female patients.

### For health extension workers

Health extension workers have to include health education during their house-to-house visit as a health education program as it positively influences TB diagnosis.

### For private health facility

Private health facilities must undergone thoroughly examination any of any patients with signs and symptoms of TB and to timely refer on time as well link them with appropriate diagnosing governmental facilities.

### For the national TB control program

National TB control programs to have to provide sufficient and basic principles of TB diagnosis for health care providers to ensure early case detection and for follow-up including the private facility to their best.

## Data Availability

All data generated or analyses during this study are included in this published article.

## Abbreviations

JBI-MAStARI: Joanna Briggs Institute Meta-Analysis of Statistics Assessment and Review Instrument
MeSH: Medical Subject Heading
NOS: Newcastle-Ottawa Scale
OR: Odds Ratio
PRISMA: Preferred Reporting Items for Systematic Reviews and Meta-Analyses
PTB: Pulmonary Tuberculosis
SDG: Sustainable Development Goals
TB: Tuberculosis
TB-SDG: Tuberculosis Sustainable Development Goals
US$: United State Dollar
WHO: World Health Organization
